# Intracerebroventricular antisense knockdown of Gα_i2 _results in ciliary stasis and ventricular dilatation in the rat

**DOI:** 10.1186/1471-2202-8-26

**Published:** 2007-04-12

**Authors:** Kati S Mönkkönen, Juhana M Hakumäki, Robert A Hirst, Riitta A Miettinen, Christopher O'Callaghan, Pekka T Männistö, Jarmo T Laitinen

**Affiliations:** 1 Department of Pharmacology & Toxicology, University of Kuopio, Kuopio, FIN-70211, Finland; 2 Department of Biomedical NMR, National Bio-NMR Facility, A.I. Virtanen Institute for Molecular Sciences, University of Kuopio, Kuopio, FIN-70211, Finland; 3 Department of Infection, Immunity and Inflammation, University of Leicester, Leicester LE2 7LX, UK; 4 Department of Neuroscience and Neurology, University of Kuopio, Finland and Department of Neurology, Kuopio University Hospital, Kuopio, FIN-70211, Finland; 5 Division of Pharmacology & Toxicology, University of Helsinki, Helsinki, FIN-00014, Finland; 6 Institute of Biomedicine, University of Kuopio, Kuopio, FIN-70211, Finland

## Abstract

**Background:**

In the CNS, the heterotrimeric G protein Gα_i2 _is a minor Gα subunit with restricted localization in the ventricular regions including the ependymal cilia. The localization of Gα_i2 _is conserved in cilia of different tissues, suggesting a particular role in ciliary function. Although studies with Gα_i2_-knockout mice have provided information on the role of this Gα subunit in peripheral tissues, its role in the CNS is largely unknown. We used intracerebroventricular (icv) antisense administration to clarify the physiological role of Gα_i2 _in the ventricular system.

**Results:**

High resolution MRI studies revealed that continuous icv-infusion of Gα_i2_-specific antisense oligonucleotide caused unilateral ventricular dilatation that was restricted to the antisense-receiving ventricle. Microscopic analysis demonstrated ependymal cell damage and loss of ependymal cilia. Attenuation of Gα_i2 _in ependymal cells was confirmed by immunohistochemistry. Ciliary beat frequency measurements on cultured ependymal cells indicated that antisense administration resulted in ciliary stasis.

**Conclusion:**

Our results establish that Gα_i2 _has an essential regulatory role in ciliary function and CSF homeostasis.

## Background

G protein-coupled receptors (GPCRs) communicate via heterotrimeric G proteins which consist of α-, β- and γ-subunits. According to the α-subunits, G proteins are divided into four classes (G_s_, G_i_, G_q _and G_12_). Half of all known α-subunits belong to the G_i_-family [1]. The need for such a multiplicity of G_i _family members is not immediately evident. Proteins of the G_i _family interact with a wide variety of GPCRs, presently considered as important drug targets.

In the CNS, the concentrations of the G_i _family subunits Gα_o _and Gα_i1 _are extremely high, and their localization in neuropil indicates involvement in neurotransmission or some other crucial functions of neuronal cells [2,3]. In contrast, Gα_i2 _has a highly restricted localization in the ventricle-surrounding areas. In the rat brain, Gα_i2 _is localized in the subventricular zone, the rostral migratory stream [4], the accessory olfactory bulb [5] and the ependymal cilia [6]. Such a specific localization implies that Gα_i2 _may well subserve physiological function distinct from those of the other Gα subunits. Moreover, Gα_i2 _is also present in motile cilia which have a characteristic 9+2 ultrastructure in different peripheral tissues, such as rat oviduct and trachea [6]. This may mean that the Gα_i2 _subunit plays a specific, regulatory role in ciliary function. These immunohistochemical findings were further supported by the proteomic analysis which revealed that Gα_i2 _is a resident axonemal protein of the human bronchial cilia [7].

It is well known that Gα_i2 _can inhibit adenylyl cyclase (AC) and thus decrease intracellular cAMP concentration [8]. Further, *in vitro *studies with Gα_i2_-knockout mice have provided information on the role of Gα_i2 _in peripheral tissues, showing Gα_i2 _to mediate neurotransmitter-dependent regulation of adenylyl cyclase in adipose tissue [9] and Ca^2+ ^channels in heart [10], as well as the signaling of the ADP receptor P2Y_12 _in platelets [11]. Gα_i2 _has also been implicated in the differentiation of hematopoietic cell lines [12], as well as in insulin signaling in the periphery [13]. Additionally, Gα_i2_-knockout studies *in vivo *have reported defects in the immune response, growth retardation and ulcerative colitis leading to premature death [14,15]. However, apart from a recent study providing evidence that a pertussis toxin-sensitive G protein, assumed to be Gα_i2_, is involved in neural stem cell proliferation in the rat subventricular zone [16], we are not aware of any reports elucidating the role of Gα_i2 _in the CNS.

In this study, we used *in vivo *antisense icv administration to clarify the physiological role of Gα_i2 _in rat brain, especially in the ependymal cilia. Although the morphology of the ciliated epithelium at the interface between the cerebrospinal fluid (CSF) and the brain parenchyma has been studied in detail [17-20], ependymal functions have remained largely unexplored. The highly restricted anatomical localization of Gα_i2 _in the ependymal cilia and the subventricular areas [4,6] makes this subtype a particularly attractive target for specific and temporally controlled knockdown by icv-delivered antisense-oligodeoxynucleotides (AS-ODNs) without the fear for development of compensatory mechanisms, which were evident in previous studies with Gα_i2 _knockout mice [9]. Our data shows that knockdown of Gα_i2 _by specific antisense oligonucleotide caused irreversible, unilateral ventricular dilatation in rat *in vivo*, and ciliary stasis on cultured rat ependymal cells *in vitro*. This suggests that Gα_i2 _has an essential regulatory role in ciliary function and CSF homeostasis.

## Results

### Icv administration of AS-ODN targeted against Gα_i2 _results in ipsilateral ventricular dilatation

**Figure 1:**
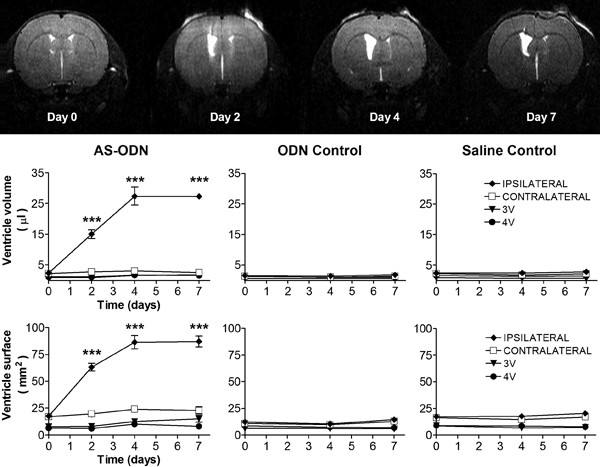
Ventricular volumes and wall surface areas imaged by high resolution MRI *in vivo*. Upper graph illustrates time-course of unilateral ventricular dilatation in AS-ODN treated rats. The brain is shown from rostral side, i.e. the targeted right lateral ventricle is on the left. The level of coronal MRI images corresponds to ~Bregma -1.0. Graphs below show ventricular volumes (μl) and wall surface areas (mm^2^) in all treatment groups calculated from consecutive MRI images using the Cavalieri principle. Data represent mean ± SEM, (n = 4–10 animals in each time point). ***, p < 0.001 significantly different from the other ipsi- and contralateral ventricles, one-way ANOVA with Tukey's Multiple Comparison test. AS-ODN, antisense-oligodeoxynucleotide against Gα_i2_; ODN control, mismatch-oligodeoxynucleotide.

**Figure 2:**
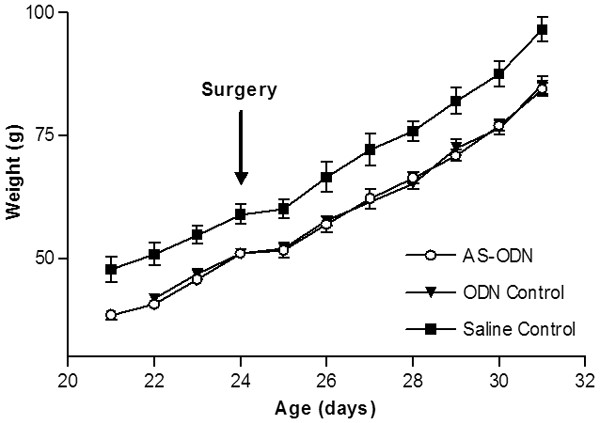
Growth curves of young (3–5 week old) animals during the 7-day infusion and observation period. Arrow shows the age of surgery (24 days) and the starting point of the icv infusion. There were no significant differences between the treatment groups in one-way ANOVA with Tukey's Multiple Comparison test. Data are mean ± SEM (n = 5–23 animals in each group in each time point).

We found that the rats receiving 7 days of icv AS-ODN treatment developed unilateral ventricular dilatation that was restricted to the AS-ODN-receiving lateral ventricle (Figure 1). Since the intracranial pressure was not assessed in this study, we call the condition ventricular dilatation instead of hydrocephalus in this text. Neither saline nor control oligodeoxynucleotides (ODN) infusion evoked a similar effect. This outcome was highly reproducible and it was evident both in young (3–5 weeks old, weighing 50–100 g), and in adult (250 g) animals. For the rest of these *in vivo *studies, young animals were used. During the 7-day infusion and observation period, the animals grew normally (Figure 2) and did not show any abnormal signs except for dehydration, which was evident from day 2 onwards. No signs of colitis or immunological abnormalities were observed. In order to evaluate the effects of AS-ODN treatment on behavior, the animals were tested for motor coordination, anxiety, motor activity as well as for pain perception and morphine analgesia. No gross alterations were observed (no significant behavioral differences between the groups, results not shown).

### Magnetic Resonance Imaging reveals the time-course for ventricular dilatation

**Figure 3:**
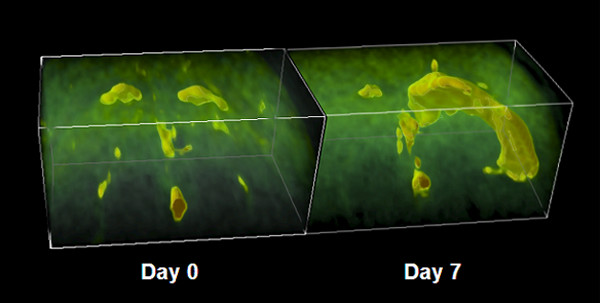
A MRI based 3D image throughout the rat ventricular system. The ventricles are viewed from caudal side, i.e. the targeted right lateral ventricle is on the right. Great enlargement of the AS-ODN receiving right lateral ventricle is seen on day 7.

The time-course of ventricular dilatation development was examined by high resolution magnetic resonance imaging (MRI) *in vivo*. The status of the ventricular system in all animal groups was assessed by MRI both prior to cannula implantation on day 0, and on days 2, 4, 7 of the icv infusion (Figures 1, 3). Ventricular volumes (μl) and wall surface areas (mm^2^) were calculated from consecutive MRI images using the Cavalieri principle [21]. The ipsilateral ventricles of AS-ODN treated rats dilated dramatically, reaching a plateau on day 4. The volume increase of the ipsilateral ventricle was statistically significant (p < 0.001) already on day 2, while the volumes of contralateral, 3rd and 4th ventricles remained constant, equal to that of day 0. Similarly, the surface area of the ipsilateral ventricle wall enlarged greatly, being significantly larger (p < 0.001) since day 2, while areas of contralateral, 3rd and 4th ventricle showed no significant changes in comparison to day 0. Animals in ODN and saline control groups exhibited no changes, either in ventricular volume or in ventricular wall surface area. Quantitative MRI data was used to create a three-dimensional model of AS-ODN animals' ventricular system as it appeared during the *in vivo *MRI study (Figure 3).

**Figure 4:**
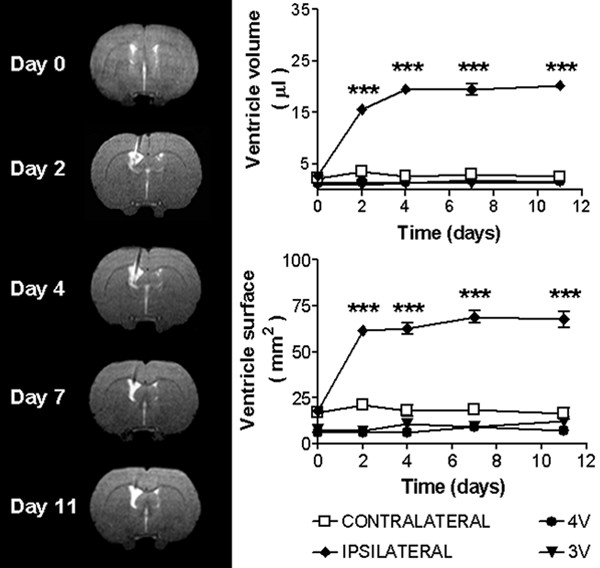
Irreversible ventricular dilatation after 2-day AS-ODN infusion, imaged by high resolution MRI *in vivo*. Left: the status of ventricular system of AS-ODN treated rats on days 0, 2, 4, 7, 11. The brain is shown from rostral side, i.e. the targeted right lateral ventricle is on the left. The level of coronal MRI images correspond to ~Bregma -1.0. Right: ventricular volumes (μl) and wall surface areas (mm^2^) calculated from consecutive MRI images using the Cavalieri principle. Data represent mean ± SEM (n = 4–5 animals in each time point). ***, p < 0.001 significantly different from the contralateral ventricle, one-way ANOVA with Tukey's Multiple Comparison test.

To clarify whether the observed phenomenon was reversible upon cessation of AS-ODN infusion, we studied the time-response relationship of 2-day icv AS-ODN treatment followed by a 9-day icv saline infusion. The dilatation of the ventricles reached a new plateau level of ventricular volume and wall surface area after the AS-ODN treatment was stopped on day 2 (Figure 4). The new plateau of volume was 28% lower, while the new plateau of the wall surface area was 23% lower than that of continuous icv AS-ODN infusion. However, the time needed to reach the new equilibrium (4 days) in ventricular volume was equal to that observed with continuous 7-day AS-ODN icv infusion. Following the 2-day AS-ODN administration, both the volume and the wall surface area in the ipsilateral ventricle were significantly (p < 0.001) larger than those of the contralateral ventricle since day 2. These experiments indicate that the AS-ODN-evoked ventricular dilatation was not reversible during the 11-day observation period.

### Histology and immunohistochemistry reveal ependymal cell damage and loss of Gα_i2_-immunoreactive ependymal cilia following AS-ODN treatment

**Figure 5:**
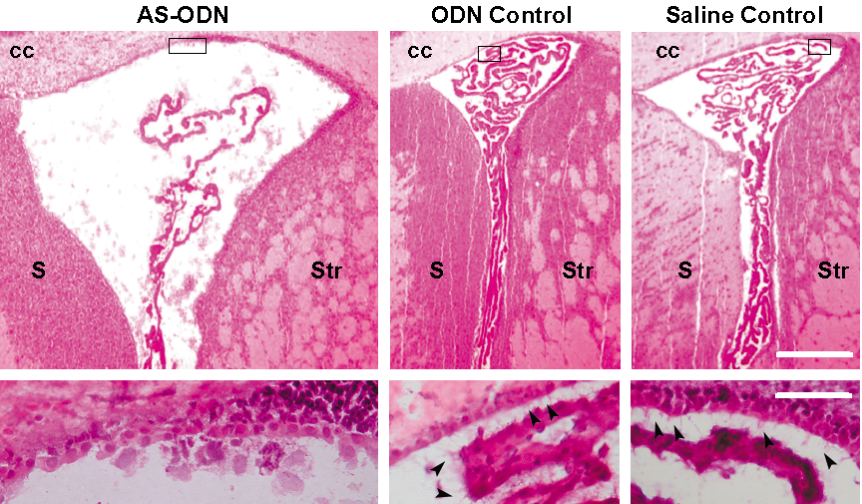
Typical light microscopic view of ipsilateral ventricles. Hematoxylin-eosin staining of coronal brain sections illustrates damaged, irregular and ruptured structure of the ependymal cell layer in AS-ODN (antisense-oligodeoxynucleotide against Gα_i2_) treated animals. Numerous dying cell populations float inside the ventricle and scarcely any cilia are present. The ependymal cell layer of ODN control (nonsense oligodeoxynucleotide) and saline control animals show a normal, uniform ependymal cell row with numerous cilia (arrowheads). Scale bar: upper images, 500 μm; lower images, 50 μm. cc: corpus callosum, S: septum, Str: striatum.

The histological status of lateral ventricles was studied in all animal groups by using hematoxylin-eosin staining. The staining was done for 8 animals in each group, and one representative specimen from each treatment group was selected to visualize the general, light microscopic view of the lateral ventricles (Figure 5). While the ependymal cell layer of ODN and saline control groups showed a normal, uniform ependymal cell row with numerous cilia, the structure of the ipsilateral ependymal cell layer in AS-ODN treated animals appeared damaged, irregular and ruptured, with practically no cilia present. Additionally, the ipsilateral ventricle of AS-ODN treated animals showed numerous dying cell populations within the ventricular cavity. Evidently, these cell populations originated from the ependymal layer as a result of massive cell damage, which followed the development of irreversible ventricular dilatation and thus, stretching of the layer. In contrast, contralateral ventricles in the AS-ODN treated animals appeared normal, identical to the ventricles of ODN and saline control animals.

**Figure 6:**
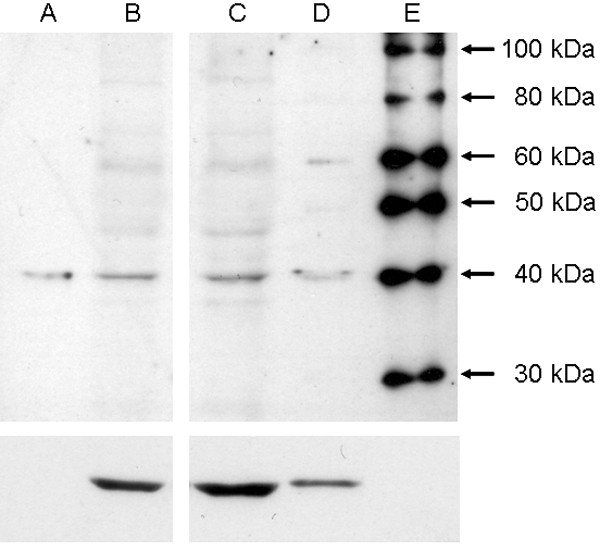
Specificity of the commercial primary antibody verified by Western blot. Upper panel: A: G protein standard, (2 μl/lane) Bovine brain immunoblot stardard, Calbiochem. B: Rat brain homogenate, (25 μg/lane). C: Rat brain homogenate, (35 μg/lane). D: Human platelet membrane (2 μg/lane). E: Molecular weight marker (MagicMark™, Invitrogen). Lower panel depicts β-actin, that was used as a loading control and was visualized after reprobing the membrane with mouse anti-β-actin antibody.

**Figure 7:**
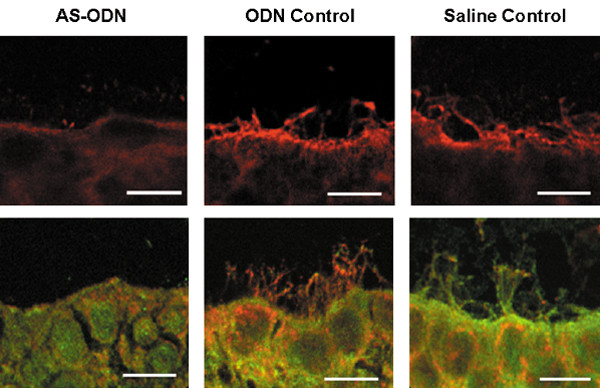
Gα_i2 _immunostaining imaged by confocal microscopy. Upper row, reference antibody against Gα_i2 _(Dr. Tomiko Asano); lower row, commercial antibody against Gα_i2 _(red) and Calbindin D-28 (green). Both antibodies show intense staining of ependymal cilia in ODN control (nonsense oligodeoxynucleotide) and saline control groups and attenuation of Gα_i2 _signal in AS-ODN (antisense-oligodeoxynucleotide against Gα_i2_) treated animals. Background staining with Calbindin D-28k (CaBP) was used to better visualize intracellular staining in endoplasmic reticulum with Chemicon antibody. Scale bar: 10 μm.

The knockdown of Gα_i2 _protein in ependymal cells was verified by immunohistochemistry using two different primary antibodies. The first was a previously validated, polyclonal affinity-purified reference antibody [22]. The second was a commercial monoclonal antibody (Chemicon), which has not been previously validated for use in immunohistochemistry. We verified the subunit specificity of this antibody by Western blotting (Figure 6). Gα_i2 _immunolabeling in ependymal cells and cilia by the two primary antibodies was imaged by confocal microscopy (Figure 7). Both antibodies showed specific and restricted labeling of Gα_i2 _in the ependymal cells and cilia while the other brain areas were immunonegative. Despite minor differences, the final outcome was the same with both antibodies. AS-ODN treated animals showed loss of ependymal cilia and attenuation of Gα_i2 _protein, whereas Gα_i2 _immunostaining in ODN and saline control groups was identical with both antibodies. While the polyclonal antibody [22] interacted solely with ependymal cilia, Chemicon's monoclonal antibody specifically recognized cilia and also labeled structures surrounding ependymal cell nucleus. The slight difference between the intracellular immunostaining with these antibodies could be due to their different target regions in the Gα_i2 _protein and/or due to potential cross-reactivity of the commercial antibody with additional targets, as evidenced by the presence of additional immunoreactive bands (~45 and ~60 kDa) in the Western blots of rat brain homogenates.

### Western blot analysis shows no alterations in Gα_i2 _immunoreactivity in more distal ventricular regions

**Figure 8:**
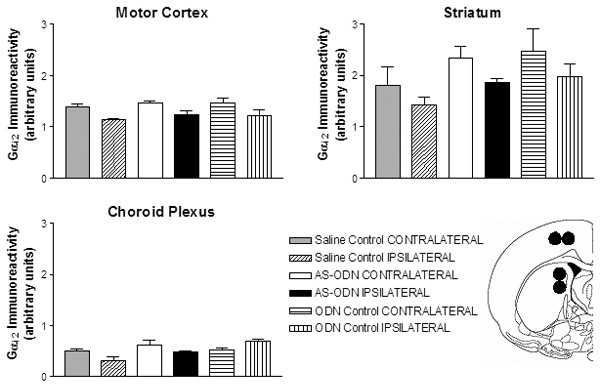
Western blot analysis of Gα_i2 _immunoreactivity in selected brain areas. Samples (diameter 1 mm) for immunoblotting were punched from 1 mm-thick brain slice as visualized in a coronal brain section. The samples of 9 animals in each group were pooled into three groups and processed in duplicate in Western blotting. Data represent mean ± SEM (n = 3). AS-ODN treated animals do not differ from control animal groups, one-way ANOVA with Tukey's Multiple Comparison test. Coronal unlabeled rat brain section (Bregma -0.26 mm) from Rat Brain Atlas [62].

To examine the extent of the AS-ODN effect, we determined Gα_i2 _levels by semi-quantitative Western blotting of selected brain structures in close vicinity to the lateral ventricles. The immunoreactivity of Gα_i2 _protein showed no changes between AS-ODN treated and control animals in motor cortex, striatum and choroid plexi (Figure 8).

### Ciliary beat frequency measurements on cultured rat ependymal cells revealed ciliary stasis following AS-ODN treatment

**Figure 9:**
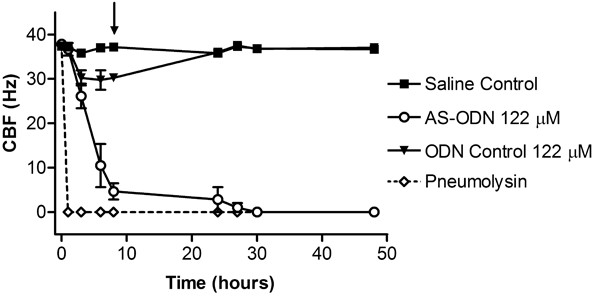
Ciliary beat frequency (CBF) measurements on cultured rat ependymal cells. Ependymal CBF over time in the presence of AS-ODN against Gα_i2 _(122 μM) and ODN control (122 μM, mismatch oligonucleotide), negative (aCSF) and positive (pneumolysin, 500 HU/ml) controls. The arrow indicates when the aCSF was exchanged for complete growth media. Data are mean ± SEM of n = 4–10. The AS-ODN (122 μM) and pneumolysin (500 HU/ml) graphs were significantly different (p < 0.001) compared to the controls, One-way ANOVA with Tukey's Multiple Comparison test.

To clarify the effect of AS-ODN on ependymal cells, we carried out ciliary beat frequency (CBF) measurements on cultured rat ependymal cells. AS-ODN administration resulted in decreased CBF, leading to irreversible ciliary stasis while the control oligo had no such effect (Figure 9). In comparison to direct toxic effect on beating cilia, namely the effect of positive control pneumolysin [23,24], the effect observed after AS-ODN administration emerged gradually, after a delay. Presumably, this delay reflects the time needed for AS-ODN to enter the cells and to terminate the synthesis of its target protein Gα_i2_. Statistical analysis showed that CBF of AS-ODN treated cells differed significantly (p < 0.001) from CBF in ODN and saline control groups.

Morphology of cultured ependymal cells treated with AS-ODN appeared normal at the timepoint when ciliary stasis occurred. However, during the 48-hour observation period the cells gradually started showing decreased viability. In contrast, cells treated with pneumolysin typically died immediately after exposure due to acute inflammation caused.

## Discussion

In this study we used *in vivo *AS-ODN administration in an effort to clarify the physiological role of Gα_i2 _in the rat ventricular system. We found that continuous icv-infusion of AS-ODN targeted against Gα_i2 _resulted in irreversible, unilateral ventricular dilatation that was restricted to the AS-receiving ventricle. High resolution MRI revealed that ventricular dilatation developed within 2 days and that the ventricle further dilated until day 4. Microscopic analysis revealed ependymal cell damage and loss of ependymal cilia. Loss of Gα_i2 _in ependymal cells was demonstrated by immunohistochemistry. CBF measurements on cultured ependymal cells indicated that AS-ODN administration resulted in ciliary stasis. Our results indicate that Gα_i2 _has an essential and previously unrecognized regulatory role in ciliary function and CSF homeostasis.

We used young, 3–5 week old male rats but the ventricular dilatation was seen after a 7-day icv AS-ODN treatment also in adult animals. Gα_i2 _protein levels in rat brain have been shown to be high already at birth, and to increase slightly until the age of 90 days [25]. Additionally, previous studies have shown that the structure of the ependymal layer reaches maturity by the age of 1–3 weeks, and that cilia are fully developed by the first postnatal week in mice and rabbits [17,19,26]. Thus, the use of young animals instead of adults was justified.

In order to avoid potential toxic effects due to the use of stable, modified oligonucleotides, we chose to use unmodified oligonucleotides which, however, are generally rapidly degraded by RNase H. In preliminary studies, we administered a single icv injection of AS-ODNs with a dose of 20 μg (in 4 μl/4 min), which, however, was without any effect (results not shown). Evidently, CSF flow constantly diluted the oligonucleotide concentration in the lateral ventricle below the effective level, and thus, continuous delivery (25 μg/day) was required to maintain an effective concentration in the target area. Similarly, siRNAs have been reported to require continuous delivery to achieve stable effects following icv administration in mice [27].

We found that continuous icv AS-ODN infusion resulted in unilateral ventricular dilatation that was restricted to the AS-ODN receiving ventricle. A detailed analysis with high resolution MRI revealed a supratentorially restricted enlargement, without any signs of subarachnoidal enlargement indicative of brain atrophy. There were no signs of asymmetry attributable to obstruction in the ventricular system, beyond the ipsilateral ventricle on MRI. In AS-ODN treated animals, neither signs of peripheral abnormalities, apart from dehydration, nor any behavioral changes were observed.

In our study, AS-ODN penetration into adjacent neural structures was not seen, as the immunoblotting of areas distant from lateral ventricles, such as motor cortex and striatum did not show any significant changes between the animal groups. Due to targeting a one cell layer with AS-ODN, immunoblotting was not an ideal method for studying Gα_i2 _knockdown in ependymal cells. Not only the quantity of these cells was very limited, especially for the immunoblotting analysis of such a low-abundance protein as Gα_i2_, but the severe damage of this cell layer in AS-ODN treated animals made it impossible to derive sufficient quantities of these cells for immunoblotting analysis. The interpretation of the results would also have been complicated, as the number of Gα_i2 _-rich cilia might have varied between the samples. In order to reliably show Gα_i2 _knockdown in each of the animals, we used immunofluorescence on rat brain cryosections, which allowed us to examine the endpoint status of individual ependymal cells and the cilia in their native environment. Moreover, this method was highly reproducible and enabled inspection of each of the animals after the treatment. While the AS-ODN treated animals showed attenuation of Gα_i2 _protein and loss of cilia, ODN and saline control groups showed identical, positive Gα_i2 _immunostaining of ependymal ciliary membrane.

The connection between ciliary beat and CSF flow direction has been evident for decades [18,28]. A recent study indicates further that beating of ependymal cilia is also required for concentration gradient formation of CSF guidance molecules directing proper migration of neuroblasts [29]. With respect to the link between cilia dysfunction and development of ventricular dilatation, it has been clearly demonstrated that ciliary dysfunction results in ventricular dilatation or hydrocephalus. Metavanadate, an inhibitor of ciliary movement, has been shown to induce hydrocephalus in rats [30]. Additionally, several animal models of congenital hydrocephalus have been reported, such as SUMS/NP mice [31] and WIC-Hyd rats [32], the latter of which have been demonstrated to exhibit immotile ependymal and respiratory cilia [33]. Both SUMS/NP and WIC-Hyd animals have a currently unknown genetic defect.

Furthermore, several studies have shown that hydrocephalus results from deficiencies in cilia structure components, such as the axonemal proteins Mdnah5 [34,35] or Spag6 [36]. Similarly, hydrocephalus has been demonstrated to follow deficiencies in proteins involved in ciliogenesis, such as Polaris [37], HFH-4 [38] and polymerase lambda [39]. Frequently, animals with cilia related genetic defects have symptoms similar to those of Primary Ciliary Dyskinesia (PCD) which occurs in humans. PCD is a condition also including Kartagener's syndrome and immotile cilia syndrome, and in addition to hydrocephalus, PCD is characterized by many accompanying symptoms such as sinopulmonary infections, situs inversus and reduced fertility [40]. This is evidence of highly conserved mechanisms in cilia motility in different tissues.

Several earlier studies have reported hydrocephalus following icv administration of viruses or bacteria [41,42]. Hydrocephalus related to local inflammation of motile cilia might result from unspecific ciliary damage and disruption of ependymal cells. However, hydrocephalus can also be induced by bacterial toxins or other inflammatory mediators causing rapid ciliary stasis, as has been shown for *mycoplasma pulmonis *[43]. There is the possibility that inhibition of ependymal cilia [24] and loss of ependymal cells [44] in pneumococcal meningitis may be directly linked with post infection hydrocephalus that is commonly observed in patients after treatment [45].

In addition to our study, there is preliminary evidence suggesting that inactivation of G_i _family proteins results in ventricular dilatation. Namely, mice with icv infection by *Bordetella pertussis *seem to develop hydrocephalus, which becomes apparent 4–6 days after the infection [46,47]. Evidently, in those early studies, hydrocephalus has developed following the inactivation of G_i _family proteins by pertussis toxin. Moreover, previous studies with cultured hamster tracheal tissues have shown that *Bordetella pertussis *infection results in ciliary stasis [48,49]. The facts that pertussis toxin induces hydrocephalus following interaction with the ependymal cilia, and causes whooping cough following interaction with the respiratory cilia indicate that the G_i _family proteins play a fundamental role in ciliary function. Given that Gα_i2 _is the predominant G_i _protein present in mammalian ependymal cilia, as well as in motile cilia in different peripheral tissues, such as rat oviduct and trachea [6,7], it may well have a ciliary-specific function. Consistent with this reasoning, we clearly showed in this study, that the knockdown of Gα_i2 _following AS-ODN treatment resulted in impaired ciliary function and resulted in unilateral ventricular dilatation *in vivo*.

In our study, microscopic analyses revealed ependymal cell damage and loss of ependymal cilia following attenuation of Gα_i2 _by AS-ODN. This outcome is in line with previous observations about ependymal injury and cell damage resulting from hydrocephalus. Following either acute or chronic hydrocephalus, the observed characteristic ependymal changes are decreased cilia density as well as a discontinuous, stretched and torn ependymal layer [50-53]. In chronic hydrocephalus, also scar formation has been reported [51]. It has been shown that irrespective of whether the hydrocephalus results from a virus, a chemical agent or a genetic defect, the ependymal changes following hydrocephalus are the same. Firstly, cilia, the most vulnerable structure, are lost, then the microvilli disappear [50], which presumably is due to extensive stretching of the ependymal layer [51]. Finally, the ependymal cells are destroyed. However, it has been demonstrated that normal cilia are present until hydrocephalus reaches a comparatively late stage [50].

In this study, we also clarified whether the ventricular dilatation was reversible at the earliest point when it was evident (day 2) but found that the phenomenon was irreversible. This outcome was expected, as it was known that in mammals, ependymal cells do not regenerate at any age [19,52]. This is largely based on the fact that proliferation markers, such as Ki-67, has not been found in the ependyma even in fetal life [54,55]. Although a regenerative response of subependymal cells following brain trauma has been reported, the resulting regeneration does not apply to the ependymal layer and furthermore, it may be subsequently overwhelmed by severe degeneration [56,57]. It is noteworthy that ciliary stasis in ependymal cell culture was irreversible as well.

Although we showed that the ventricular dilatation observed as an endpoint condition in our study could be explained by ciliary stasis following AS-ODN treatment, our study can not establish a causal link between the two events *in vivo*. We cannot totally exclude the fact that ciliary immobility may not have been the only factor in the development of ventricular dilatation. Although Gα_i2 _appears not to be particularly enriched in the choroid plexus (our unpublished observations), knockdown of Gα_i2 _in this tissue might also have contributed to the disturbed CSF homeostasis and ventricular dilatation. This reasoning is relevant in light of previous findings showing that intracellular cAMP levels positively regulate CSF production in the choroid plexus [58]. Besides this, a recent study suggested that elevated activity of cAMP pathway might be involved in development of hydrocephalus, as elevated PKA and PKC activity was shown to result in ciliary defects and development of hydrocephalus in mice overexpressing PAC1 receptor [59]. Further studies will be required to rule out these and the other possible factors, e.g. local inflammation, in the development of ventricular dilatation following AS-ODN treatment.

Interestingly, dehydration, which was highly repeatable in our study after 2 days of continuous icv AS-ODN infusion, was also reported in a study with icv *Bordetella pertussis *infection in mice [46]. Given the peculiar localization of the circumventricular organs (CVOs) bordering the 3rd and 4th ventricle, it seems possible that CSF might have a role in their neuroendocrine regulatory functions [60]. It is clear that AS-ODNs delivered intracerebroventricularly can readily reach CVOs and thus, might easily interfere with the signalling pathways regulating body fluid homeostasis. Previously, it was demonstrated that *in vivo *icv administration of an AS-ODN targeted against the angiotensin AT_1 _receptor into 3rd ventricle resulted in thirst attenuation in rats, indicating that icv AS-ODN treatment can effectively reach CVO cells [61].

## Conclusion

The aim of this study was to clarify the physiological role of Gα_i2 _in the ependymal cilia by silencing its function by *in vivo *icv antisense oligodeoxynucleotide (AS-ODN) administration. Our results show that AS-ODN knockdown of Gα_i2 _resulted in irreversible, unilateral ventricular dilatation in rat *in vivo*, and ciliary stasis on cultured rat ependymal cells *in vitro*, raising the possibility that ciliary stasis resulted in ventricular dilatation *in vivo*. Collectively these findings indicate that Gα_i2 _has a hitherto unrecognized physiological role in the regulation of ependymal ciliary function and CSF homeostasis in the ventricular cavity. To the best of our knowledge, this is a novel finding and therefore may have immediate relevance to the neuroscience community.

## Methods

### Animals

Three weeks old male Harlan Wistar rats (total n = 71 for *in vivo *studies) were used in accordance with the European Communities Council Directive (86/609/EEC) and following a protocol approved by the local ethics committee. Animals were bred in National Laboratory Animal Center, University of Kuopio, and by National Public Health Institute, Kuopio, and housed five per cage in 12-h light/12-h dark cycle (lights on at 07:00 h) with food and water ad libitum. In studies with adult animals, 250 g weighing male Wistar rats were used. In ependymal cell culture we used newborn Wistar rats (younger than 24 h), bred by Biomedical Services, University of Leicester, UK.

### Stereotaxic surgery

Animals were assigned in three groups (saline control, AS-ODN and ODN control) and after three days of adaptation to their home cages, at the age of 24 days, brain infusion cannulas were placed in right lateral ventricles under chloral hydrate (Merck, Darmstadt, Germany) anesthesia (350 mg/kg i.p.) using stereotaxic surgery. Anatomical structures were identified based on Rat Brain Atlas [62]. Cannulas were fixed in the skull with metal or nylon screws (Plastics One, Roanoke, VA) and dental cement (Voco, Cuxhaven, Germany). The Alzet minipumps (Models 1007D and 2002, Durect, Cupertino, CA) were sited subcutaneously dorsally. After cannula placement, rats were housed one per cage to prevent damage of the wounds and tubing. Animals received saline (n = 22), antisense (n = 27), nonsense (n = 17) or mismatch (n = 5) oligonucleotides (Sigma-Genosys, Haverhill, UK) as continuous icv infusion for 7 days (25 μg/day) starting from surgery (days 24–31). Animals were examined and weighed daily to ensure health and growth. The same procedures were used on adult rats (250 g, total n = 5).

### Oligonucleotides

Sequences for unmodified 18-mer oligonucleotides were chosen based on GenBank database search. The sequence against Gα_i2 _did not match any other rat sequence except for the N-terminal sequence of Gα_i2 _(nucleotides 150–167). Selected nonsense and mismatch sequences did not match any rat sequence. The following sequences were used: anti-Gα_i2_: 5'-CTT GTC GAT CAT CTT AGA-3', nonsense: 5'-GGG GGA AGT AGG TCT TGG-3', and mismatch 5'-TCT GCT GAT ACT CTT GAA-3' which was used as a more stringent control, maintaining equal base composition to the antisense molecule but with eight mismatches. The HPLC-purified (purity 90–97%) oligonucleotides were dissolved in sterile saline. RNase free, aseptic conditions were maintained until implantation of the cannulas to prevent degradation of unmodified oligonucleotides by RNase H prior to reaching the target cells.

### Behavioral studies

On the 7th day of continuous icv infusion, animals (n = 8 per group) were tested for motor coordination, anxiety, motor activity as well as for pain perception and morphine analgesia. In rota-rod test, the ability of rats to stay on revolving rod (2 rpm) was examined for 1 min. Elevated plus maze measured anxiety and consisted of a plus-shaped maze with two open and two enclosed arms. The rats were individually placed into the maze for 3 min and their movements and the time spent in all of the arms was determined. Normally, rats prefer the enclosed arms but show interest to enter also the open arms. Anxious animals tend to remain in the enclosed arms. The open field test measured motor activity and consisted of a circular maze with radial and circular lines marked on the floor. The rats were individually placed into the maze and the motor activity (line crossing) was observed for 2 min. In the tail immersion test, the outermost 4 cm portion of the rat tail tip was immersed in +53°C water bath 28 minutes after s.c. morphine injection (10 mg/kg). The reaction time of tail withdrawal was measured. The test was terminated after 15 s if the rat gave no response to this slightly painful stimulus.

### Magnetic resonance imaging

To assess the status of the lateral, 3rd and 4th ventricles *in vivo*, animals were examined by high resolution magnetic resonance imaging (MRI). For this purpose, saline control, ODN control (mismatch oligonucleotide), and AS-ODN groups were studied (n = 5 per group) before implantation of infusion cannulas (day 0) as well as during the infusion (days 2–7). Prior to MRI studies, rats were anesthetized with chloral hydrate (350 mg/kg i.p.), immobilized, and externally fixed to a custom-built animal holder. To maintain normal body temperature, heated air was blown through the magnet bore during the study. MRI was performed using A SMIS console (Surrey Medical Imaging Systems, Guildford, UK) interfaced to a 9.4 T vertical magnet (Oxford Instruments, Oxford, UK). A single loop surface coil (diameter 27 mm) was used in transmit/receive mode. Multi-slice T_2_-weighted images were acquired using a single-echo spin-echo method (time-to-repetition 2000 ms, time-to-echo 40 ms, 4 averages/line, matrix size of 256 × 128 (zerofilled to 256 × 256), field of view (25.6 mm × 25.6 mm) and 20 slices at slice thickness of 0.7 mm). Brain ventricle volumes (μl) and ventricle wall surface areas (mm^2^) were calculated from consecutive images by the Cavalieri principle [21]. Animals were imaged immediately prior to implantation of cannulas by stereotaxic surgery (day 0), followed by MRI studies 2 (antisense rats only), 4, and 7 days later during continuous icv infusion.

### Histology

On the 7th day of continuous icv infusion, the rats (n = 8 per group) were decapitated and the whole brains were dissected out, dipped in isopentane on dry ice and stored at -80°C. Serial coronal 20 μm-sections were cut at -20°C using a Reichert-Jung cryostat, approximately spanning Bregma 1.2 mm to -1.8 mm [62]. Six coronal sections, each from an individual animal and two from each treatment group, were collected per slide and thaw-mounted onto Super-Frost*/Plus slides (Menzel-Gläser, Braunschweig, Germany) at 20°C. The sections were stored at -80°C. Routine hematoxylin-eosin staining was used to identify possible damage of the brain. For immunohistochemistry, slides with brain sections were thawed by immersion (10 min at 20°C) into fixative containing 4% paraformaldehyde (TAAB, Berkshire, UK) and 0.05% glutaraldehyde (TAAB, Berkshire, UK) in 0.1 M phosphate buffer (PB), pH 7.4. The slides were then washed for 2 × 5 min with 0.1 M phosphate buffered saline (PBS), the excess buffer was removed and nonspecific binding was blocked by 1.5% normal horse serum (NHS) for 15 minutes. Following removal of excess NHS, the slides were incubated with the primary antibodies [Chemicon slides: 1:1000 Mouse anti-Gα_i_-2 monoclonal antibody, MAB3077 (Chemicon International, Temecula, CA); and 1:1000 Rabbit anti-CaBP, CB 38 (Swant, Bellinzona, Switzerland); Asano slides: 1 μg/ml Rabbit anti-Gα_i2_, a kind gift from Dr. Tomiko Asano] or with 0.1 M PB, pH 7.4 (negative controls) overnight at 4°C. The slides were washed with 0.1 M PB, pH 7.4 for 5 min and incubated with secondary antibodies [Chemicon slides: 1:100 Biotinylated horse anti-mouse (BA-2000, Vector Laboratories, Peterborough, UK); and 1:300 Goat anti-rabbit Alexa Fluor 488 (Molecular Probes, Eugene, OR); Asano slides: 1:2000 Goat anti-rabbit-Cy5 (Jackson ImmunoResearch, West Grove, PA)] for 30 min at 20°C. The slides with biotinylated secondary antibodies were then incubated with 1:50 streptavidin Cy-5 (Jackson ImmunoResearch, West Grove, PA) for 40 min at 20°C. Following antibody incubations, all slides were washed for 2 × 5 min with 0.1 M PB, pH 7.4 and rinsed with water prior to coverslipping with Gelmount (Biomeda, Foster City, CA). All the washing steps and the dilutions of the antibodies were carried out in 0.1 M PB, pH 7.4. The immunofluorescent slides were imaged using Nikon Eclipse-TE300 inverted microscope, (Nikon Corporation, Japan) with Ultra VIEW laser scanning confocal device (Perkin-Elmer, UK) using 60× objective. Images were further processed using Adobe Photoshop (Adobe Systems, Mountain View, CA).

### Western blotting

Within each treatment group (n = 9), the animals were assigned in groups of three to pool tissues for Western blotting. 1-mm coronal slice from frozen brains (Bregma 1.00 – 0.00 mm) were cut and two punches of 1 mm diameter were taken from each slice from left and right frontal motor cortex (mCtx) and striatum (Str). Rest of the brain was thawed in ice-water bath and the choroid plexi (ChP) were dissected out from both lateral ventricles. Pooled tissues were sonicated in sonication buffer (1 mM EDTA, 20 mM Tris-HCl pH 7.5) for 5 × 1 sec (mCtx, Str) or for 10 × 1 sec (ChP) in an ice-water bath. The protein content of the sonicates was measured using bicinchoninic acid (BCA) protein assay by Pierce. Sonicates (35 μg protein/lane) were resolved in 10% SDS-PAGE and blotted onto nitrocellulose membrane. Nonspecific binding was blocked by 5% non-fat dry milk in Tris-buffered saline containing 0.05% of Tween 20 (TTBS). Blots were incubated overnight in primary antibody [1: 500 Mouse anti-Gα_i_-2 monoclonal antibody, MAB3077 (Chemicon International, Temecula, CA)] in 2.5% non-fat dry milk in TTBS at 4°C. After 4 × 10 min washes in TTBS the blots were incubated in secondary antibody [1: 4000 Goat anti-mouse IgG-HRP, SAB-100 (StressGen Biotechnologies, San Diego, CA)] in 2.5% non-fat dry milk in TTBS for 1 h at 20°C. Blots were washed 4 × 10 min with TTBS and the chemiluminescent reaction was started with Western Lightning chemiluminescent reagents (NEL104, NEL105, Perkin-Elmer Life Sciences, Inc. Boston, MA) and the signal was detected on BioMax Light Film (Cat 1788207, Kodak, Rochester, NY). To detect β-actin, the blots were incubated in stripping buffer (in 100 mM β-mercaptoethanol, 2% SDS and 62.5 mM Tris-HCl, pH 6.7) for 30 min at + 50°C and non-specific binding was blocked by 5% non-fat dry milk in TTBS. Blots were incubated overnight in primary antibody [1: 1000 Mouse monoclonal anti-β-actin, clone AC-15, ascites fluid A5441 (Sigma-Aldrich, St Louis, MO)] in 0.2% BSA and 0.03% NaN_3 _in Tris-buffered saline followed by 4 × 10 min washes in TTBS. Finally, blots were incubated with secondary antibody [1: 20 000 Goat anti-mouse IgG-HRP, SAB-100 (StressGen Biotechnologies, San Diego, CA)] in 2.5% non-fat dry milk in TTBS for 1 h at 20°C. The pooled sonicates in all treatment groups were processed as duplicates in Western blot. Rat forebrain punch sonicate was used as an internal standard. Rat brain homogenate and human platelet membranes were used to verify the subunit specificity of the primary antibodies in Western blotting. The films were scanned using HP Precision Scan Pro and the band densities were quantified in proportion to densities of β-actin and internal standard bands by using Scion Image software (Scion Corporation, Fredrick, MD).

### Ependymal Cell Culture

Ependymal cells were grown using a method adapted from Wiebel and colleagues [23,63]. Eight-well (25 × 35 mm) tissue culture trays were coated with bovine fibronectin (75 μg/2 ml; ≅ 1 μg/cm^2^) and incubated at 37°C in 5% CO_2 _for 24 hours prior to use. New-born (younger than 24 hours old) Wistar rats were killed by cervical dislocation and their brains removed using careful dissection. The cerebellum was removed, as was small (3 mm) edge regions of the frontal cortex and the left and right cortical hemispheres. The remaining brain regions (containing ependymal cells and progenitors) were mechanically dissociated by passing the tissue through an 18-gauge needle in 2 ml of tissue culture medium. The complete growth medium was serum-free Minimum Essential Medium (Gibco Life Technologies, Paisley, Scotland) containing penicillin (100 IU/ml), streptomycin (100 μg/ml), fungizone (2.5 μg/ml), bovine serum albumin (5 μg/ml), insulin (5 μg/ml), transferrin (10 μg/ml), selenium (5 μg/ml) and from day 3 onwards thrombin (0.5 IU/ml). Dissociated tissue from a single brain was seeded (500 μl per well) into eight well (25 × 35 mm) tissue culture trays, with each well containing 2 ml of medium. The medium was replaced 3 days after seeding. The adherent ependymal cells were fed by the replacement of 2 ml of fresh medium two times a week. The ciliated ependymal cell colonies were identified at day 5 and experiments were done using cells that were between 14–28 days old, a time when cell proliferation was optimal.

### Oligonucleotide addition

All experiments were performed in filter sterilised (0.2 μm, acrodisc) 1 ml of artifical cerebrospinal fluid [aCSF (mM): NaCl (128), KCl (3), CaCl_2_(1.3), MgCl_2 _(1), NaH_2_PO_4 _(22.3), D-Glucose (1.25) in deionised water pH 7.4 with 10 M NaOH]. Pneumolysin has previously been shown to inhibit CBF in ependymal cultures [23], therefore it was used throughout as a positive control. Pneumolysin was purified as previously described [64], made up in aCSF at 500 Haemolytic Units (HU)/1 ml and used as a positive control for inhibition of ependymal ciliary beat frequency (CBF). Negative controls were incubated in 1 ml of aCSF alone. AS-ODN and ODN control oligonucleotides were mixed with 1 ml of aCSF at 122 μM then added to the cells. The cells were incubated at (37°C) for 8 hours with CBF readings taken at 0, 1, 3, 6 and 8 hours. At 8 hours the aCSF was exchanged for 1 ml of complete growth medium and incubated at 37°C for 48 hours with CBF readings taken at 24, 27, 30 and 48 hours.

### Ciliary beat frequency

To determine ciliary beat frequency the cells were placed in a humidified (70–90%) incubation (37°C) chamber and observed using an inverted microscope system (Diphot, Nikon, UK). Beating cilia were recorded using a digital high-speed video camera (Kodak Ektapro Motion Analyser, Model 1012) at a rate of 400 frames per second, using a shutter speed of 1 in 2000. The camera allows video sequences to be recorded and played back at reduced frame rates or frame by frame. Ciliary beat frequency was determined by timing a given number of individual cilia beat cycles. Basal ciliary beat frequency was measured at 0 minutes, before addition of oligonucleotides or pneumolysin. Calculation of CBF (Hz): 400 (Number of frames per second)/5 (frames elapsed for 5 ciliary beat cycles) X 5 (conversion per beat cycle).

### Data analysis

The MRI results are presented as mean ± SEM. CBF data are presented as mean ± SEM of 12–30 individual CBF measurements from 4–10 separate cell cultures. Mean reading of each individual culture was obtained from 3 randomly chosen cilia. Statistical comparison was performed by using one-way analysis of variance with Tukey's multiple comparison test, analyzed with GraphPad Prism for Windows (GraphPad Software, Inc., San Diego, CA). Statistical significance was achieved when p < 0.05.

## Abbreviations

AC, adenylyl cyclase; aCSF, artificial cerebrospinal fluid; AS-ODN, antisense-oligodeoxynucleotide; CaBP, calbindin D-28k; CBF, ciliary beat frequency; ChP, choroid plexus; CSF, cerebrospinal fluid; CVOs, circumventricular organs; GPCRs, G protein-coupled receptors; icv, intracerebroventricular; IFT, intraflagellar transport; mCtx, motor cortex; MRI, magnetic resonance imaging; NHS, normal horse serum; ODN, oligodeoxynucleotide; PCD, Primary Ciliary Dyskinesia; PB, phosphate buffer; PBS, phosphate buffered saline; Str, striatum; TTBS, Tris buffered saline containing 0.05% Tween 20.

## Authors' contributions

KSM carried out the animal procedures, including stereotaxic surgery and behavioral experiments, performed histological and Western blot analyses, participated in magnetic resonance imaging and design of the study and drafted the manuscript. JMH designed and carried out the magnetic resonance imaging studies, RAH designed and carried out the ciliary beat frequency studies including ependymal cell culture and data analysis, RAM designed and participated in the histological studies, COC designed ciliary beat frequency studies including ependymal cell culture, PTM designed the behavioral animal studies and participated in design of the study, JTL conceived of the study, its design and coordination. All the authors read and approved the final manuscript.
